# Motor function and safety of nusinersen for spinal muscular atrophy: a systematic review and meta-analysis among Chinese patients

**DOI:** 10.3389/fped.2026.1767428

**Published:** 2026-03-13

**Authors:** Guanlan He, Zilu Feng, Na Li, Shasha Wei, Yunyuan Liu, Xiuneng Tang

**Affiliations:** 1 Department of Clinical Pharmacy, Maternal and Child Health Hospital of Guangxi Zhuang Autonomous Region, Nanning, China; 2 School of Pharmacy, Guangxi Medical University, Nanning, China

**Keywords:** China, meta-analysis, motor function, nusinersen, safety, spinal muscular atrophy, systematic review

## Abstract

**Background:**

This study aimed to perform a systematic review and meta-analysis to evaluate motor function and safety outcomes in Chinese patients with spinal muscular atrophy (SMA) treated with nusinersen.

**Methods:**

We searched across multiple databases, including PubMed, Cochrane, Embase, Web of Science, CNKI, CBM, Wanfang, and VIP, for real-world studies (RWS) published before 15 February 2026. Data extraction is performed based on predefined study selection and eligibility criteria, while excluding studies involving non-Chinese populations.

**Results:**

24 RWS were included in this analysis, comprising 1,273 Chinese patients with SMA Types 1–4 treated with nusinersen. Statistically significant improvements were found in the Children's Hospital of Philadelphia Infant Test of Neuromuscular Disorders (CHOP-INTEND) [Mean Difference (MD) = 6.57 (95% CI: 4.35–8.80)], Hammersmith Infant Neurological Examination Section 2 (HINE-2) [MD = 2.05 (95% CI: 0.94–3.16)], Hammersmith Functional Motor Scale Expanded (HFMSE) [MD = 4.71 (95% CI: 3.10–6.32)], Revised Upper Limb Module (RULM) [MD = 3.70 (95% CI: 2.47–4.93)], and the 6-minute walk test (6MWT) [MD = 24.96 m (95% CI: 16.20–33.72)] at the end of the follow-up period. In terms of clinical response rate, CHOP-INTEND (≥4 points) was 71.80% (95%CI: 56.14%–85.65%), HFMSE (≥3 points) was 59.71%(95%CI: 43.79%–74.74%), RULM(≥2 points) was 60.35% (95%CI:48.67%–71.51%), respectively. As treatment duration increased, motor function scores and clinical response rates either improved or stabilized. When the treatment duration reached ≥14 months, the pooled mean change from baseline was as follows: CHOP-INTEND [MD = 9.45 (95% CI: 5.03–13.87)], HFMSE [MD = 4.37 (95% CI: 3.34–5.40)]], and RULM [MD = 2.83 (95% CI: 1.97–3.68)]. Clinical response rates were: 76.41% (95% CI: 52.89%–94.57%) for CHOP-INTEND, 69.83% (95% CI: 48.60%–87.61%) for HFMSE, and 63.23% (95% CI: 45.86%–79.13%) for RULM. The overall adverse event (AE) rate was 41.27% (95% CI: 29.65%–53.38%), with serious AEs occurring in 1.26% (95% CI: 0%–5.85%) of Chinese patients. The most common AEs were headache, back pain, and post-lumbar puncture syndrome, associated with the lumbar puncture administration method.

**Conclusion:**

Our meta-analysis indicates that nusinersen is associated with a statistically significant and clinically meaningful improvement in motor function among Chinese patients with types 1–4 SMA in real-world studies. The reported adverse events align with the expected safety profile of nusinersen. However, the majority of studies included in this review had a follow-up period of less than 24 months. Therefore, further long-term follow-up is necessary to assess the sustained therapeutic effects of nusinersen in Chinese patients with SMA.

**Systematic Review Registration:**

https://www.crd.york.ac.uk/PROSPERO/view/CRD42023403580, PROSPERO CRD42023403580.

## 1 Introduction

Spinal muscular atrophy(SMA) is a rare, severe autosomal recessive neuromuscular disorder characterized by progressive muscle weakness (1). It typically manifests with early-onset hypotonia and motor dysfunction, which can lead to secondary complications affecting the skeletal, respiratory, and gastrointestinal systems. Respiratory insufficiency, resulting from progressive involvement of the respiratory muscles, is the primary cause of mortality in individuals with SMA (1, 2). SMA also is a leading genetic cause of infant mortality, with an incidence of approximately 8 per 100,000 live births (3).The most common cause of SMA is a homozygous deletion or point mutation in the survival motor neuron 1 (SMN1) gene, located on chromosome 5q13. This genetic mutation leads to decreased levels of the SMN protein, resulting in the degeneration of α-motor neurons and progressive muscular atrophy (4, 5).

SMA is categorized into five subtypes based on age of onset and maximal motor function achieved: type 0–4 (1). SMA type 0, which has the earliest age of onset and the most severe clinical presentation, is characterized by profound muscle weakness and hypotonia at birth, with affected infants typically succumbing within weeks. SMA type 1 patients exhibit symptoms within 6 months of age and never achieve the ability to sit independently. SMA type 1 is characterized by symptom onset before 6 months of age, with affected individuals never achieving the ability to sit independently. Patients with type 2 SMA experience symptom onset between 6 and 18 months of age and are generally able to sit independently but are unable to walk unaided. In type 3 SMA, symptoms manifest after 18 months of age, and patients initially attain the ability to walk independently, though this ability may be lost over time. Type 4 SMA presents in adulthood, with mild or minimal symptoms emerging later in life (6–8).

In recent years, the introduction of disease-modifying therapies (DMTs), including nusinersen, risdiplam, and onasemnogene abeparvovec, has significantly improved the prognosis for patients with SMA. Nusinersen, the first antisense oligonucleotide DMT for SMA, was approved by the U.S. Food and Drug Administration (FDA) in 2016. Onasemnogene abeparvovec and risdiplam were subsequently approved by the FDA in 2019 and 2020, respectively.

Nusinersen, currently recognized as the preferred disease-modifying therapy for SMA across all patient age groups and subtypes, has primarily been evaluated in non-Chinese populations in previous clinical trials. There had been a limited number of studies compared to other countries, with the approved of Nusinersen in China, and more and more real-world studies (RWS) about Chinese SMA population in recent years. However, these observational studies generally exhibit methodological variations, considerable heterogeneity, and inconsistent findings. A meta-analysis was performed to systematically synthesize and assess informative to draw more precise and reliable conclusions regarding the motor function outcomes and safety of nusinersen in Chinese patients with SMA types 1–4 under RWS.

## 2 Methods

### 2.1 Protocol registration

This meta analysis was conducted in accordance with the Preferred Reporting Items for Systematic Reviews and Meta-Analysis (PRISMA) guidelines, along with the methodological principles outlined in the Cochrane Collaboration Handbook (9). The protocol was registered on the International Prospective Register of Systematic Reviews (PROSPERO) (ID: CRD 42023403580).

### 2.2 Information sources, search, eligibility criteria and data extraction

A systematic search was conducted to retrieve published literature from PubMed, Web of Science, Embase, Cochrane Library, China National Knowledge Infrastructure (CNKI), Chinese Biomedical Literature Database (CBM), WanFang Database andWeiPu (VIP) Databases up to 15 February, 2026. We used combinations of the following keywords for the literature search: “spinal muscular Atrophy”, “SMA”, “Oligonucleotides, Antisense”, “nusinersen”,“Spinraza”, “treatment outcome”, “safety”, “efficacy”. No search restriction was applied. Reference lists from both included studies and relevant reviews were examined to identify additional studies.

The following inclusion criteria were used in this study: (1) Randomized controlled trials (RCTs), case-control studies, cohort studies (prospective or retrospective), or case series were considered for inclusion; (2) The study participants were Chinese patients with SMA who received treatment with nusinersen; (3) The study clearly reported the administration method and dosage of the treatment medication. Combinatorial treatment of other DMT drugs, Cross-sectional studies, case report, and review articles were excluded. Studies that reported a comparison between baseline and post-treatment outcomes for the same population were included.

The outcome indicators included at least one of the following: (1) adverse events; (2) motor function scales, including the Children's Hospital of Philadelphia Infant Test of Neuromuscular Disorders (CHOP-INTEND), Hammersmith Infant Neurological Examination Section 2 (HINE-2), Hammersmith Functional Motor Scale Expanded (HFMSE), Revised Upper Limb Module (RULM), and 6-minute walk test (6MWT) (10, 11); (3) the incidence of patients achieving clinical improvement in any of the primary outcomes(clinical response rate), based on previous reports. Clinical response rate was defined as an increment of at least 4 points from baseline for the CHOP-INTEND score, 2 points for HINE-2, 3 points for the HFMSE score, 2 points for RULM, and 30 meters in the 6MWT (10–13).

Study selection and eligibility criteria were defined based on the PICOS framework (populations, interventions, comparators, outcomes, and study design). Two independent researchers (Guanlan He and Zilu Feng) screened the titles and abstracts, followed by a full-text review to assess eligibility based on the predefined criteria. For RWS, the following data were extracted: study type, age, gender, sample size, SMA type, follow-up duration, and research outcomes. In case of a disagreement, the researchers discussed the issue or consulted a third reviewer until a consensus was reached.

### 2.3 Quality assessment

To ensure that the findings of the review were based on the best available evidence, the Newcastle-Ottawa Scale (NOS), a systematic and comprehensive quality assessment tool, was used to evaluate the quality of the included studies for meta-analysis (14). The NOS is a widely used quality assessment tool for observational studies, particularly suitable for case-control and cohort studies. Studies with scores of 1–4 were considered poor quality, 5–7 were considered of moderate quality, and 8–9 were considered of high quality.

### 2.4 Statistical analysis

Data were extracted from all studies that met the inclusion criteria for meta-analysis. Both quantitative meta-analyses and descriptive analyses were performed for each category, including motor function, clinical response rate, and adverse event rate. Studies were included in the meta-analysis if they (1) reported the mean change and corresponding standard deviation (SD) for CHOP-INTEND, HINE-2, HFMSE, RULM, and 6MWT scores from baseline, defined as nusinersen treatment initiation to a specified time point (e.g., 2, 6, 10, or ≥14 months from baseline), along with the clinical response rate for each indicator and the incidence of adverse events, or (2) provided accessible individual patient data sufficient to calculate the required summary statistics. Case report involving only a single patient, which precluded the calculation of SD, were excluded from meta-analysis.

Stata 17.0 was used for the analysis. The primary outcome of interest regarding pre- and post-treatment comparisons of motor function scales, was assessed using effect sizes (ES). Continuous variables were reported as mean difference (MD) with a 95% confidence interval (CI) (15). The clinical response rate and adverse events were expressed as incidence rates (P) with 95% CI (16). For the original data that did not conform to a normal distribution, a double arcsine transformation was applied to stabilize the variance of the original ratio (15, 16).

Heterogeneity among the included studies was assessed using the Q-test and I^2^ statistics to quantify the extent. A Q-test *p* value < 0.10 or I^2^ > 50% indicated significant heterogeneity of findings among studies. Considering the heterogeneous nature in RWS, random effects models were performed in meta-analyses (6, 17).

## 3 Results

### 3.1 Study selection and basic characteristics

**Figure 1:**
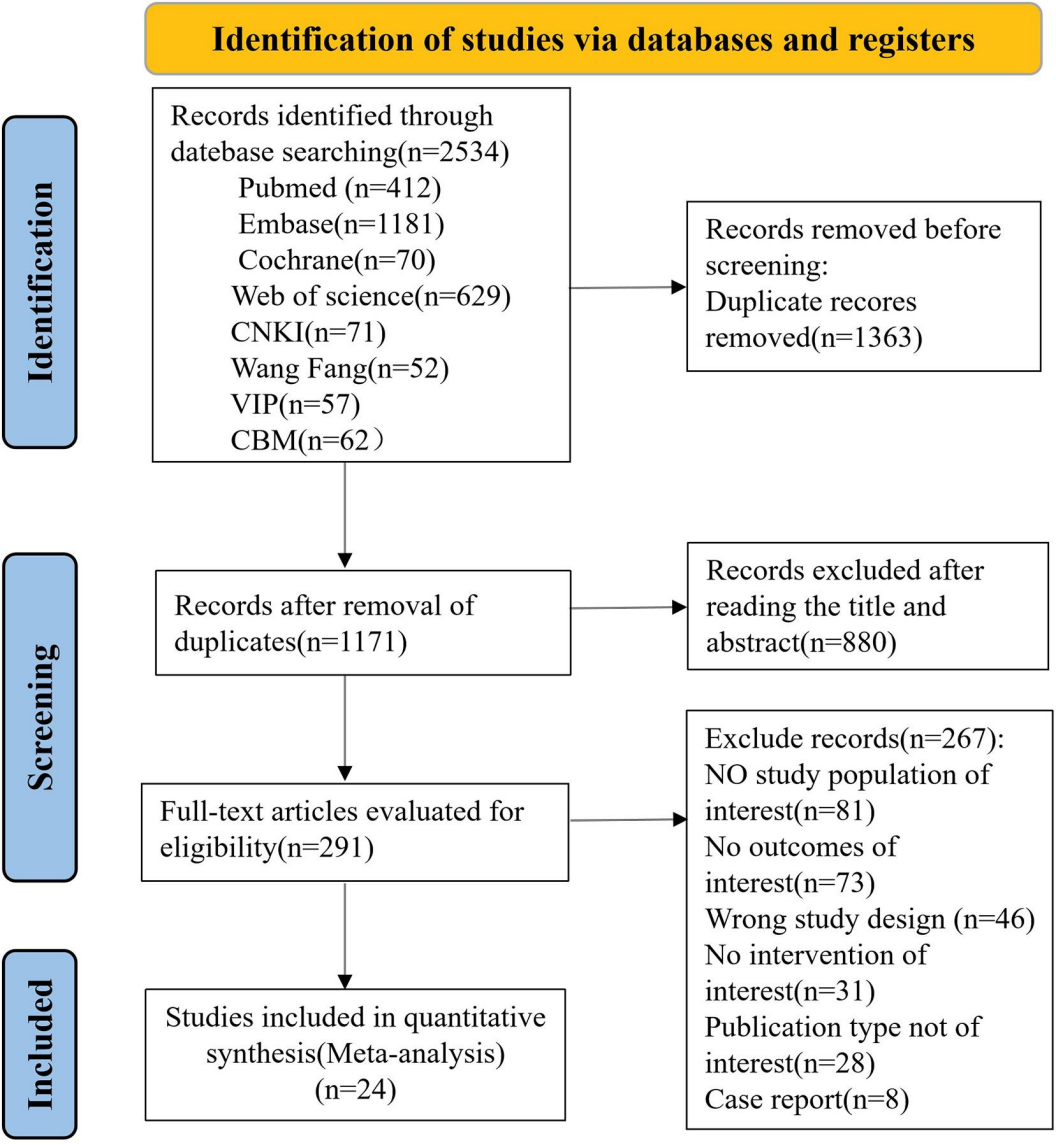
The PRISMA flow diagram illustrating the selection process of studies included in this systematic review.

A total of 2,534 records were identified through our database search. After removing 1,363 duplicates, the titles and abstracts of 1,171 articles were screened.Following this, 880 records were excluded, leaving 291 studies for full-text review. Of these, 267 studies were eliminated after full-text assessment. Finally, 24 articles involving 1,273 Chinese patients with SMA types 1–4 were included in this review. Figure 1 describes the search and selection process under the PRISMA flow diagram (9). The baseline characteristics of the included studies are summarized in Table 1.

**Table 1:** The baseline characteristics of the included studies for meta-analysis.

First author/year	Study type	Centers	Total participants	SMA type	*SMN2* copy number	Age at treatment (year, MD ± SD, Median, range)	Follow Up (months)	Outcome
Shanshan Mao 2022 (18)	Retrospective cohort	Single center	15	1,2,3	Not Reported	6.8 (2.8,8.3)	9–24	②③④⑤⑥
Jin Guo 2024 (19)	Retrospective cohort	Single center	50	1,2,3	2: 7 3: 38 4: 5	5.0 (2.5,9.3)	6–22	①②④⑤
Yiwen Zhu 2023 (20)	Retrospective cohort	Single center	19	1,2,3	Not reported	7.2 (1.6,17.5)	10	①③④⑤
Kai Ma 2023 (21)	Retrospective case series	Single center	68	1,2,3	2: 6 3: 49 4: 11 Unknow: 2	2.7 ± 1.5	≥14	①②④⑤⑥
Dandan Xu 2024 (22)	Retrospective cohort	Single center	22	2,3	Not reported	6.8 ± 3.5	≥12	④⑤
Yuyi Chen 2024 (23)	Retrospective cohort	Single center	52	1,2,3	2: 4 3: 46 4: 2	3.7 (0.4,16)	6–14	②④⑤
Xiaoli Yao 2024 (24)	Prospective cohort	Multicenter	385	1,2,3	2: 41 3: 225 ≥ 4: 26 unknow: 96	6.3 ± 5.1	6–14	①②③④⑤
Hoi Ning Hayley Ip 2024 (25)	Prospective cohort	Multicenter	23	1,2,3	2: 5 3: 14 4: 4	5.8 (0.4,17.5)	6–12	②③④⑤⑥
Qiaoyan Shao 2023 (26)	Prospective cohort	Single center	42	1,2,3	Not reported	6.8 ± 3.9	10–24	①②④⑤
Yu Chen 2023 (27)	Retrospective case series	Single center	6	3,4	Not reported	29.0 ± 10.9	2–6	①
Hongyan Shen 2024 (28)	Retrospective case series	Single center	5	Not reported	Not reported	6（1.25,14）	2–24	②
Yunsong Li 2025 (29)	Retrospective cohort	Single center	18	1,2,3	2: 5 3: 10 4: 3	7.1 (0.3,15.0)	12–18	①④⑤⑥
Yuwu Jiang 2024 (30)	Prospective cohort	Multicenter	50	Infantile-onse, later-onset	2: 7 3: 32 ≥ 4: 3 Unknow: 8	4.6 (1,21)	2–18	①③
Liyuan Chen 2023 (31)	Retrospective cohort	Single center	18	2,3	3: 15 4: 3	9.8 ± 4.0	2	②③④⑤⑥
Yang Huang 2025 (32)	Prospective cohort	Single center	28	1,2	Not reported	7.43 ± 3.2	6	④
Li Wenjing 2025 (33)	Retrospective cohort	Single center	42	1,2,3	Not reported	4.4 ± 4.1	2–18	①②③④⑤
Sihui Chen 2025 (34)	Prospective cohort	Single center	28	3,4	2: 11 3: 16 4: 1	25.3 ± 8.9	18	①④⑤⑥
Xiaoying Wang 2025 (35)	Retrospective cohort	Single center	27	1,2,3	Not reported	5.6 ± 5.4	14	①
Jihua Wu 2025 (36)	Retrospective cohort	Single center	61	1,2,3	2: 7 3: 54	4.3 ± 2.6	14	①②④⑤⑥
Bing Wang 2023 (37)	Prospective cohort	Single center	29	2,3,4	2: 3 3: 10 4: 9 Unknow: 7	22 (14,52)	6–10	①④⑤⑥
Yingshuang Peng 2025 (38)	Prospective cohort	Single center	41	1,2,3	2: 2 3: 28 4: 1 Unknow: 10	4.6 ± 2.6	6–14	①②③④⑤
Yijie Feng 2025 (39)	Retrospective cohort	Single center	50	2,3	2: 4 3: 46	5.84 (3.42,9.11)	6–34	①④⑤
Xinzhu Liu 2025 (40)	Retrospective cohort	Single center	22	1,2,3	2: 3 3: 18 4: 1	13.2 (9.8–21.8)	≥2	①⑤
Yi Dai 2025 (41)	Prospective and Retrospective cohort	Multicenter	172	1,2,3,4	2: 7 3: 89 4: 69 Unknow: 7	26 (20,33)	14	①②④⑤⑥

① adverse events; ② CHOP-INTEND; ③ HINE-2; ④ HFMSE; ⑤ RULM; ⑥ 6MWT.

### 3.2 Quality assessment of included studies

Based on the NOS quality assessment of the studies included in the meta-analysis, four studies received a score of 8, ten studies received a score of 7, eight studies received a score of 6, and two studies received a score of 5, indicating that the quality of the included studies was acceptable. The detailed results are presented in Table 2. Since all the included studies were single-arm RWS, the comparability category was rated as low. Considering the general limitations of RWS in rare diseases, such as limited sample size and difficulty in observing a nonexposed cohort within the same study, the quality of these studies was relatively high and acceptable.

**Table 2:** Newcastle–Ottawa scale quality assessment of studies included in the meta-analyses.

Study	Selection	Comparability	Exposure/outcome	Overall score
Shanshan Mao 2022 (18)	3	1	3	7
Jin Guo 2024 (19)	3	1	3	7
Yiwen Zhu 2023 (20)	3	1	3	7
Kai Ma 2023 (21)	2	1	3	6
Dandan Xu 2024 (22)	3	1	3	7
Yuyi Chen 2024 (23)	3	1	3	7
Xiaoli Yao 2024 (24)	3	1	3	7
Hoi Ning Hayley Ip 2024 (25)	3	1	3	7
Qiaoyan Shao 2023 (26)	2	1	3	6
Yu Chen 2023 (27)	2	1	3	6
Hongyan Shen 2024 (28)	2	1	2	5
Yunsong Li 2025 (29)	3	1	3	7
Yuwu Jiang 2024 (30)	3	1	2	6
Liyuan Chen 2023 (31)	2	1	3	6
Yang Huang 2025 (32)	4	1	3	8
Li Wenjing 2025 (33)	4	1	3	8
Sihui Chen 2025 (34)	3	1	3	7
Xiaoying Wang 2025 (35)	2	1	3	6
Jihua Wu 2025 (36)	2	1	3	6
Bing Wang 2023 (37)	2	1	3	6
Yingshuang Peng 2025 (38)	4	1	3	8
Yijie Feng 2025 (39)	4	1	3	8
Xinzhu Liu 2025 (40)	2	1	2	5
Yi Dai 2025 (41)	3	1	3	7

### 3.3 Motor function

#### 3.3.1 CHOP-INTEND

**Figure 2:**
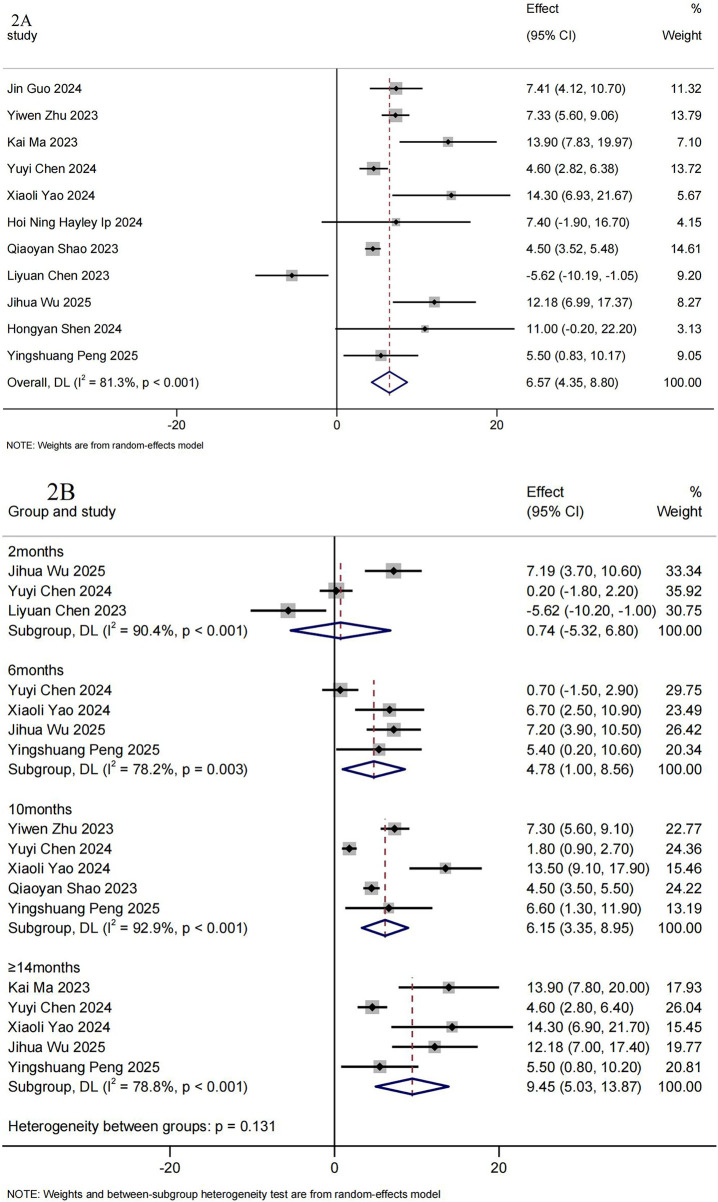
Summary of meta-analysis for CHOP-INTEND. **(A)** Mean CHOP-INTEND differences from baseline. **(B)** Mean CHOP-INTEND differences from baseline after treatment with Nusinersen for 2, 6, 10 and ≥14 months.

11 RWS with CHOP-INTEND outcome were included in the meta-analysis, involved 109 infants or non-sitters with SMA Type 1–2 (19–21, 23–26, 28, 31, 36, 38). The pooled mean change from baseline on CHOP-INTEND was 6.57 (95%CI: 4.35–8.80) (Figure 2A). Figure 2B summarizes the pooled mean change from baseline and the corresponding 95% CIs from each model in the meta-analysis. The mean increase in CHOP-INTEND scores was 0.74 (95%CI: −5.32–6.80) at 2 months (*n* = 34) (14, 23, 28), 4.78 (95%CI: 1.00–8.56) at 6 months (*n* = 54) (23, 24, 36, 38), 6.15 (95%CI: 3.35–8.95) at 10 months (*n* = 46) (20, 23, 24, 26, 38), 9.45 (95%CI: 5.03–13.87) at ≥14 months (*n* = 74) (21, 23, 24, 36, 38) in the overall population.

10 RWS (*n* = 104) (18–21, 23, 25, 26, 28, 33, 36) reported that the percentage of patients considered to have a clinically meaningful CHOP-INTEND response (≥ 4 points) at the end of the follow-up period was 71.80% (95%CI: 56.14%–85.65%). As the treatment duration increased, the clinical response rate further increased, the clinical response rate further improved and tended to stabilize after 10 months of treatment with nusinersen. The percentage of patients with a clinically meaningful CHOP-INTEND response (≥ 4 points) was 37.30% (95%CI: 18.25%–58.32%) at 2 months (23, 36)^,^ increasing to 58.56% (95%CI: 37.13%–78.67%) at 6 months (23, 24, 36, 38), 77.46% (95%CI: 39.64%–100.00%) at 10 months (23, 24, 26, 38), and 76.41% (95%CI: 52.89%–95.47%) at ≥14 months (21, 23, 24, 36, 38) (Table 3).

**Table 3:** Clinical response rate.

Project	Number of included studies	Sample size	*P*	*I* ^2^	Effect model	Response rate(95%CI)
CHOP-INTEND	10	104	0.07	43.70	Random	71.80%(56.14%–85.65%)
2 months	2	24	<0.01	0.00	Random	37.30%(18.25%–58.32%)
6 months	4	36	0.23	30.80	Random	58.56%(37.13%%-78.67%)
10 months	4	19	0.10	51.20	Random	77.46%(39.64%–100.00%)
≥14 months	5	44	0.10	47.80	Random	76.41%(52.89%–94.57%)
HFMSE	12	355	<0.01	87.94	Random	59.71%(43.79%–74.74%)
2 months	3	99	0.25	27.10	Random	36.43%(25.29%–48.31%)
6 months	6	306	0.39	4.76	Random	54.74%(48.76%–60.66%)
10 months	6	210	0.57	0.00	Random	55.35%(48.36%–62.23%)
≥14 months	6	183	<0.01	87.86	Random	69.83%(48.60%–87.61%)
RULM	12	307	<0.01	74.12	Random	60.35%(48.67%–71.51%)
2 months	3	82	0.13	51.80	Random	51.04%(35.15%–66.83%)
6 months	6	274	<0.01	73.58	Random	48.74%(36.06%–61.50%)
10 months	6	184	<0.01	70.78	Random	47.91%(33.45%–62.54%)
≥14 months	6	140	<0.01	74.45	Random	63.23%(45.86%–79.13%)
6MWT ≥ 30 m	5	49	<0.01	83.90	Random	61.87%(20.55%–96.12%)

#### 3.3.2 HFMSE

**Figure 3:**
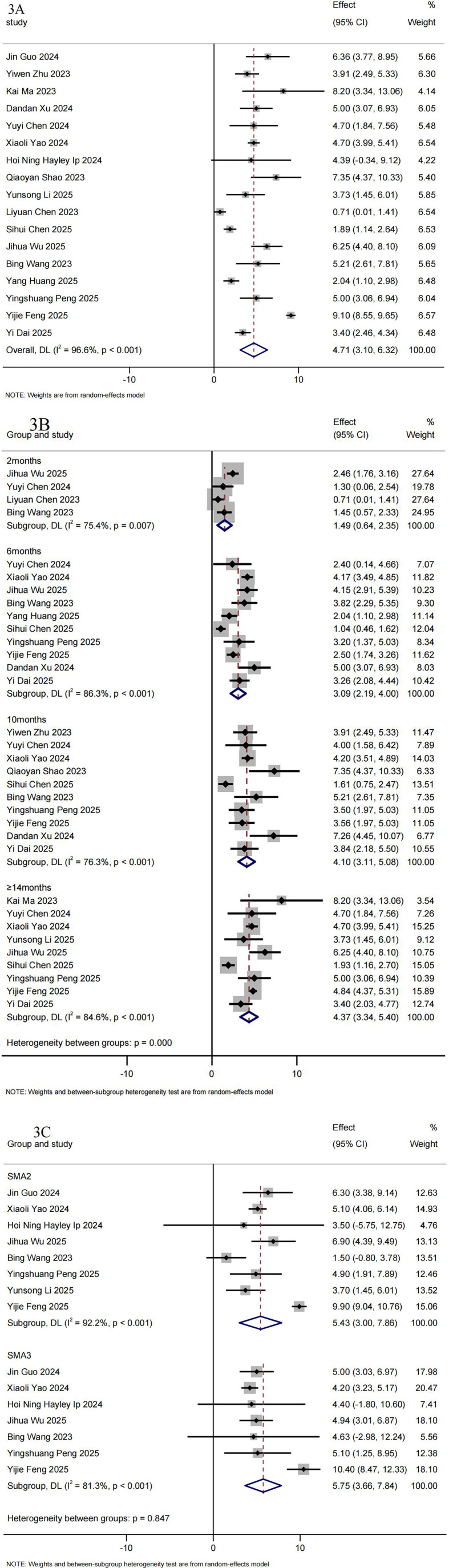
Summary of meta-analysis for HFMSE. **(A)** Mean HFMSE scores differences from baseline. **(B)** Mean HFMSE scores differences from baseline after treatment with Nusinersen for 2, 6, 10 and ≥14 months. **(C)** Mean HFMSE scores differences from baseline in SMA2 and SMA3 Chinese patients.

A total of 17 RWS (19–26, 29, 31, 32, 34, 36–39, 41) involving 721 patients with SMA type 2–4 reported HFMSE results. The pooled mean change from baseline on HFMSE was 4.71 (95%CI: 3.10–6.32) (Figure 3A). Figure 3B summarizes the pooled mean change and the 95%CI from the baseline to the follow-up time in the meta-analysis. The mean increase in HFMSE scores was 1.49 (95%CI: 0.64–2.35) at 2 months (*n* = 125), 3.09 (95%CI: 2.19–4.00) at 6 months (*n* = 535), 4.10 (95%CI: 3.11–5.08) at 10 months (*n* = 503), 4.37 (95%CI: 3.34–5.40) at ≥14 months (*n* = 496) in the overall population (Figure 3B).

12 RWS (*n* = 355) (18–21, 23, 25, 29, 32–34, 36, 41) reported changes in HFMSE scores pre- and post-treatment with nusinersen. The overall clinical response rate for HFMSE score (≥3 points) during the follow-up period was 59.71% (95%CI: 43.79%–74.74%). As the treatment duration increased, the clinical response rate of nusinersen therapy continued to improve. The percentage of patients with a clinically meaningful HFMSE response was 36.43% (95%CI: 25.29%–48.31%) at 2 months (*n* = 99), increasing to 54.74% (95%CI: 48.76%–60.66%) at 6 months (*n* = 306), 55.35%(95%CI: 48.36%–62.23%) at 10 months (*n* = 240), and 69.83% (95%CI: 48.60%- 87.61%) at ≥14 months (*n* = 183) (Table 3).

The magnitude of the increase in HFMSE scores from baseline was greater among patients with SMA Type 3 than those with Type 2 at the end of the follow-up period (Figure 3C). The pooled mean change from baseline on HFMSE in Chinese patients with SMA type 2 was 5.43 (95%CI: 3.00–7.86) (*n* = 249). While in SMA type 3, it was 5.75 (95%CI: 3.66–7.84) (*n* = 182). At ≥14 months, the mean increase in HFMSE scores was 4.92 (95%CI: 4.32–5.51) for Type 2 and 5.01 (95%CI: 3.95–6.07) for Type 3. Detailed forest plots for RULM scores by SMA subtype are provided in supplementary appendix Supplementary Figures S1,S2.

#### 3.3.3 RULM

**Figure 4:**
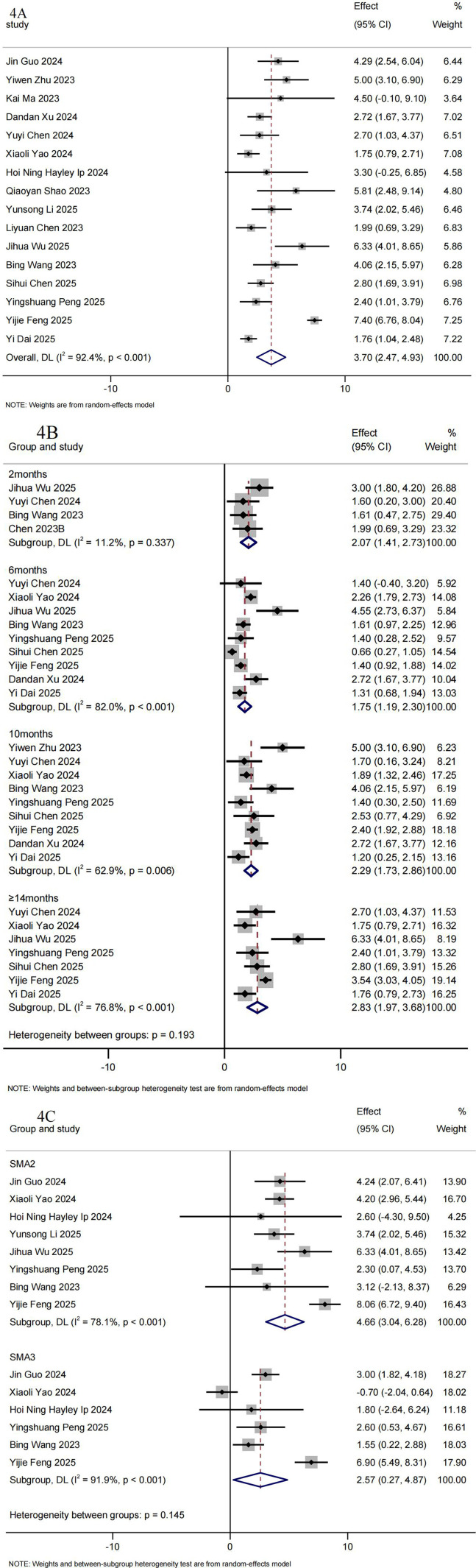
Summary of meta-analysis for RULM. **(A)** Mean RULM scores differences from baseline. **(B)** Mean RULM scores differences from baseline after treatment with Nusinersen for 2, 6, 10 and ≥14 months. **(C)** Mean RULM scores differences from baseline in SMA2 and SMA3 Chinese patients.

A total of 16 RWS (19–26, 29, 31, 34, 36–39, 41) including 662 Chinese patients with SMA type 2–4 reported RULM results. The pooled mean change from baseline on RULM was 3.70 (95%CI: 2.47–4.93) (Figure 4A). Statistically significant increases from baseline in RULM scores were consistently observed at each time point. Figure 4B summarizes the pooled mean change and the 95%CI from baseline to follow-up in the meta-analysis. The mean increase in RULM scores was 2.07 (95%CI: 1.41–2.73) at 2 months (*n* = 114), 1.75 (95% CI: 1.19–2.30) at 6 months (*n* = 440), 2.29 (95%CI: 1.73–2.86) at 10 months (*n* = 406), 2.83 (95%CI: 1.97–3.68) at ≥14 months (*n* = 373) in the overall population (Figure 4B).

12 RWS (*n* = 307) (18–21, 23, 29, 31, 33, 34, 36, 40, 41)reported changes in RULM scores pre- and post-treatment with nusinersen. The overall clinical response rate for RULM scores (≥2 points) during the follow-up period was 60.35% (95%CI:48.67%–71.51%). As the treatment duration increased, clinical response rate of nusinersen therapy continued to improve. The percentage of patients with a clinically meaningful RULM response (≥ 2 points) was 51.04% (95%CI: 35.15%–66.83%) at 2 months (*n* = 82), increasing to 48.74% (95%CI: 36.06%–61.50%)at 6 months (*n* = 274), 47.91% (95%CI: 33.45%–62.54%) at 10 months (*n* = 184), and 63.23% (95%CI: 45.86%–79.13%) at ≥14 months (*n* = 140) (Table 3).

The magnitude of the increase in RULM scores from baseline was greater among patients with SMA Type 2 than those with Type 3 at the end of the follow-up period (Figure 4C). The pooled mean change from baseline on RULM in Chinese patients with SMA 2 Type was 4.66 (95%CI: 3.04–6.28) (*n* = 207), while in SMA Type 3, it was 2.57 (95%CI: 0.27–4.87) (*n* = 150). At ≥14 months, the mean increase in RULM scores was 4.01 (95%CI: 3.04–6.28) for Type 2 and 1.83 (95%CI: −1.08–4.75) for Type 3. Detailed forest plots for RULM scores by SMA subtype are provided in supplementary appendix Figs.S3-S4.

#### 3.3.4 HINE-2

**Figure 5:**
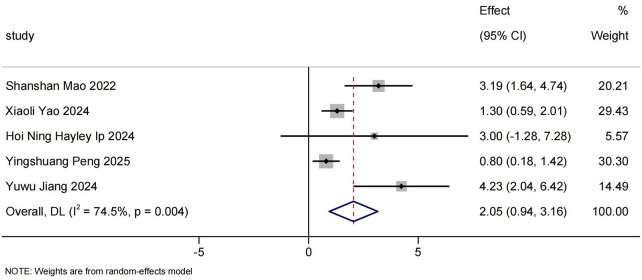
Mean HINE-2 scores differences from baseline.

Analysis of HINE-2 score was conducted in 5 RWS with a total of 91 SMA Type 1 patients (18, 24, 25, 30, 38).The pooled mean change from baseline on HINE-2 was 2.05 (95%CI: 0.94–3.16) (Figure 5).

Only two studies reported the percentage of patients considered to have a clinically meaningful HINE-2 response (≥ 2 points) at the end of the follow-up period. One study (Shanshan Mao 2022) reported that all Chinese SMA patients (*n* = 15) achieved a clinically significant improvement in motor function on the HINE-2 score after treatment with nusinersen (≥ 2 points) (18). Another study (Li Wenjing 2025) reported that the clinical response rate was 55.56% (33).

#### 3.3.5 6MWT

**Figure 6:**
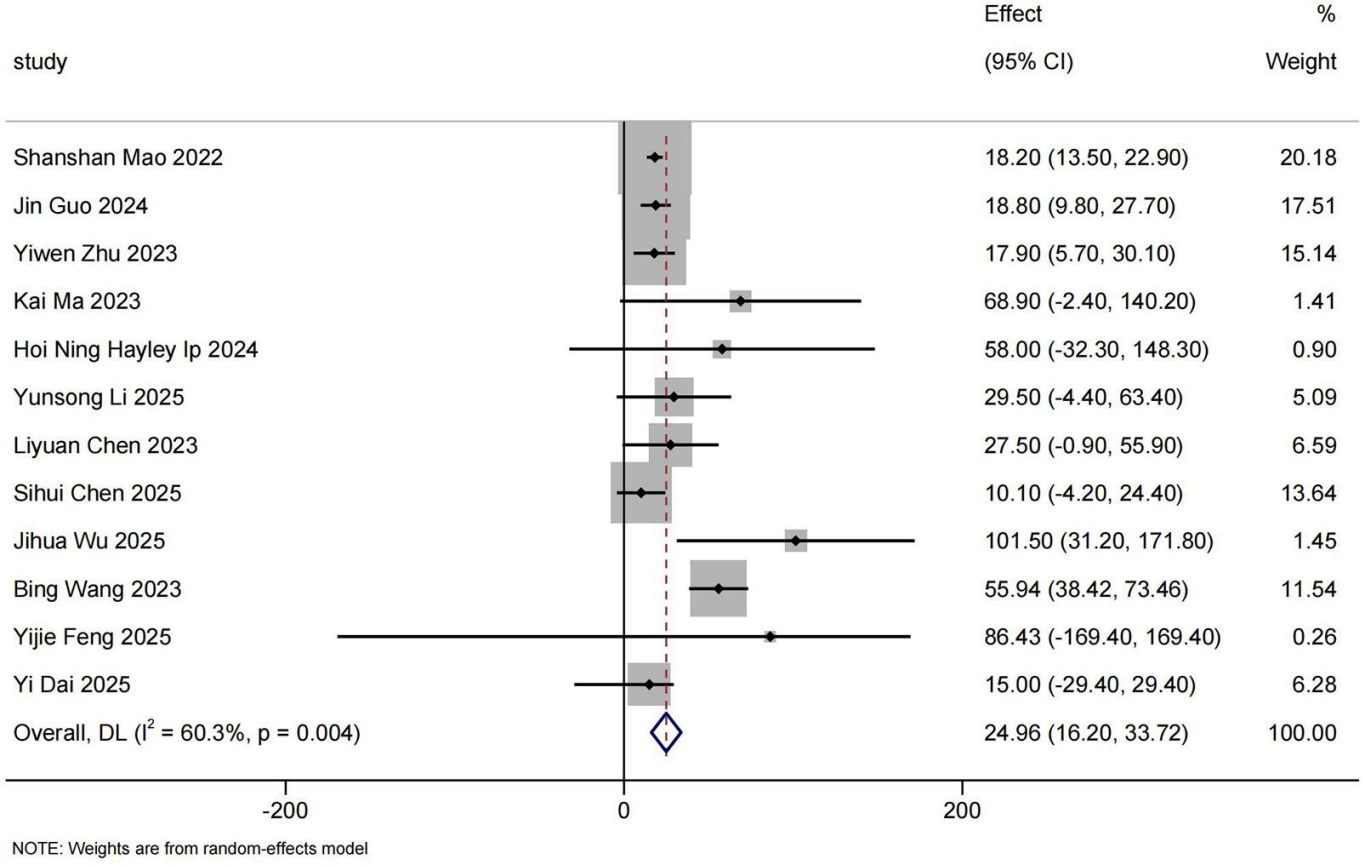
Mean 6MWT scores differences from baseline.

A total of 12 RWS reported 6MWT results (18–21, 25, 29, 31, 34, 36, 37, 39, 41), including 127 SMA Type 3 Chinese walkers. The pooled mean change from baseline on 6MWT was 24.96 m (95%CI: 16.20–33.72) (Figure 6).

5 RWS (*n* = 49) (21, 31, 36, 39, 41) reported that the percentage of patients considered to have a clinically meaningful 6MWT response (≥ 30 m) at the end of the follow-up period was 61.87% (95%CI: 20.55%–96.12%). Sihui Chen 2025 (34) reported that 6 patients (37.50%) experienced an increase of more than 20 m in the 6WMT during the follow-up period.

### 3.4 Safety

16 RWS (19–21, 24, 26, 27, 29, 30, 33–35, 37–41) reported data on total adverse events (AEs) and severe adverse events (SAEs) from 657 Chinese patients. Most of the adverse events that occurred during nusinersen treatment were related to the primary SMA disease and its related complications. Only one study (Xiaoli Yao 2024) (24) reported the rate of nusinersen-related adverse events, which was 1.51%.The overall rate of AEs was 41.27% (95% CI: 29.65%–53.38%), while the rate of SAEs was 1.26% (95% CI: 0.00%–5.85%). Among specific adverse events, headache was the most common at 15.17% (95% CI: 6.85%–25.61%), followed by back pain at 13.92% (95% CI: 9.30%–19.19%), and post-lumbar puncture yndrome (PLPS) at 9.59% (95% CI: 2.19%–20.63%), Which is associated with the lumbar puncture administration method. Other specific adverse events included nausea at 11.27% (95% CI: 2.05%–25.33%), respiratory tract infection 8.82%(95% CI: 2.96%–16.95%), fever 8.01% (95% CI: 3.36%–14.11%), abnormal liver function 9.00% (95% CI: 0.00%–27.95%), pneumonia 7.95% (95% CI: 1.67%–17.58%), vertigo 7.93% (95% CI: 3.06%–14.47%), vomiting 5.78% (95% CI: 3.29%–8.79%), respiratory distress 5.44% (95% CI: 2.37%–9.51%), COVID-19 4.92% (95% CI: 2.33%–8.27%), diarrhea 4.90%, hematuresis3.79%, cough 3.29%, and abdominal pain 2.80% (Table 4).

**Table 4:** Adverse events rates.

AES	Number of included studies	Sample size	*P*	*I*^2^/%	Effect of model	R%(95%CI)
Overall rate of AEs	16	657	<0.01	88.63	Random	41.27%(29.65%–53.38%)
Rate of SAEs	6	334	0.01	69.14	Random	1.26%(0–5.85%)
Headache	11	338	<0.01	79.97	Random	15.17%(6.85%–25.61%)
Back pain	11	465	0.04	46.87	Random	13.92%(9.30%–19.19%)
Nausea	5	167	<0.01	80.74	Random	11.27%(2.05–25.33)
Respiratory tract infection	7	395	<0.01	80.30	Random	8.82%(2.96%–16.95%)
Fever	8	324	0.01	62.92	Random	8.01%(3.36%–14.11%)
Post-lumbar puncture syndrome	4	243	<0.01	78.12	Random	9.59%(2.19%–20.63%)
Abnormal liver function	4	119	<0.01	79.59	Random	9.00%(0–27.95%)
Pneumonia	4	277	<0.01	80.01	Random	7.95%(1.67%–17.58%)
Vertigo	2	97	<0.01	0.00	Random	7.93%(3.06%–14.47%)
Vomiting	10	345	<0.01	0.71	Random	5.78%(3.29%–8.79%)
COVID-19	3	232	0.54	0.00	Random	4.92%(2.33%–8.27%)
Respiratory distress	2	174	<0.01	0.00	Random	5.44%(2.37%–9.51%)
Diarrhea	3	118	0.13	0.98	Random	4.90%(0.28%–13.01%)
Cough	4	187	0.27	22.85	Random	3.29%(0.66%–7.29%)
Hematuresis	2	100	<0.01	0.00	Random	3.79%(0.62%–8.79%)
Abdominal pain	2	69	<0.01	0.00	Random	2.80%(0–8.73%)

## 4 Discussion

Previous clinical trials of DMTs for SMA were primarily conducted in non-Chinese populations. However, with the launch of nusinersen in China and its inclusion in the national medical insurance, the number of RWS targeting the Chinese population has gradually increased, particularly in the past two years. This study systematically analyzed and integrated RWS on nusinersen for Chinese SMA patients, demonstrating that nusinersen significantly improves motor function in many Chinese patients with SMA types 1–4.

SMA is a rare genetic disorder. Prior to the introduction of nusinersen, the lack of effective therapeutic drugs and intervention measures, means that management for SMA patients primarily focused on symptomatic control and palliative care. In the long-term natural history follow-up, motor function in untreated children with SMA type 1 was severely delayed, with clinical response rates for CHOP-INTEND and HINE-2 assessments approaching zero by 12 months of age (42–44).

Although research indicates that some young patients with SMA types 2 and 3 maintain relatively stable motor function, lung function, and muscle mass within the first 12 months (45, 46). However, long-term follow-up studies have demonstrated that untreated patients with SMA types 2 and 3 typically experience a gradual decline in motor function. This decline is reflected in varying degrees of reduction in scores such as the HFMSE, RULM, and 6MWT (47–51). Such progressive deterioration in untreated individuals may significantly impact their ability to perform activities of daily living.

Nusinersen is an antisense oligonucleotide that increases the production of functional SMN protein by targeting the SMN2 gene. It is administered via spinal injections and is recommended for all patients with confirmed presymptomatic SMA or types 1, 2, 3 and 4 SMA (42, 52, 53). A growing body of research indicates that treatment with nusinersen may offer significant clinical benefits in improving motor function in patients with SMA. Our meta-analysis results were consistent with those of other studies (6, 54, 55), demonstrating improved motor function in Chinese patients with SMA type 2–4 treated with nusinersen. During the follow-up period of the included studies, we observed a pooled mean improvement from baseline in HFMSE [4.71 (95% CI: 3.10–6.32)], RULM [3.70 (95%CI: 2.47–4.93)], and 6MWT [24.96 m (95%CI: 16.20–33.72)] in Chinese patients with SMA types 2–4. In the subgroup analysis based on follow-up time duration, we found that changes in HFMSE scores increased with longer treatment durations. The magnitude of the increase from baseline in HFMSE scores was slightly greater among patients with SMA Type 3 than Type 2 across all timepoints. RULM scores showed a temporary decline at 6 months but increased at 10-month and ≥14 months follow-up intervals. In contrast to the pattern observed for HFMSE, the magnitude of increase in RULM scores was greater for patients with SMA Type 2 than Type 3. The results of the analysis of HFMSE and RULM scores in Chinese patients with SMA type 2 and 3 were similar to those of Tim Hagenacker (54).

We also examined clinically meaningful response rate after nusinersen treatment. Patients with treatment of nusinersen in type 2 and 3 Chinese patients had high response rates: 54.74% (27.3, 77.9) for HFMSE and 48.74% for RULM at 6 months, 55.35% for HFMSE and 47.91% for RULM at 10 months. Similar clinical response rates for HFMSE and slightly lower response rates for RULM were reported in this analysis compared with the meta-analysis conducted by Xinran Zhao et al. (6), which reported rates of 53.0% and 54.4% for HFMSE and RULM over 6 months, respectively. This observation may be attributed to differences in the timing of outcome assessments and populations, such as age, SMN2 copy unmber. Our meta-analysis also reported clinical response rates of 69.83% for HFMSE and 63.23% for RULM, based on a follow-up period at 14 months or longer.

With the accumulation of RWS over the past two years, we also reported changes in CHOP INTEND scores of Chinese patients with SMA Type 1 and 2 who were unable to sit independently before receiving nusinersen treatment, in comparison with their baseline levels. Our meta-analysis indicate that, without considering follow-up time, the CHOP INTEND score in Chinese SMA patients demonstrated an increase of 6.57 (95% CI: 4.35–8.80) compared to baseline, with a clinical response rate of 71.80% (95% CI: 56.14%–85.65%). In the subgroup analysis based on follow-up duration,we observed that as treatment duration increased, both CHOP INTEND score and clinical response rate improved further. Clinical response rate tended to stabilize after 10 months of treatment. Compared with the meta-analysis by Coratti et al. (55), Our meta-analysis included a longer follow-up period. The study by Coratti et al. included only two articles with a 12-month follow-up period and conducted a qualitative descriptive analysis, without performing a quantitative synthesis. HINE-2 score is primarily used to assess the development of children between 2 months and 2 years. Our findings show that, without differentiating follow-up time, the HINE-2 score increased by 2.05 (95% CI: 0.94–3.16) compared to baseline, indicating significant improvement.

In our meta-analysis, the overall AEs rate and SAEs rate in Chinese SMA patients treated with nusinersen were 41.27% and 1.26%, respectively. Compared to the study by Zhijuan Zhong et al. (56), the incidence of adverse events was significantly lower. However, most adverse events were related to the primary SMA disease. The adverse reactions directly associated with nusinersen accounted for 1.15%, with aseptic encephalitis being the most commonly reported, though only one study reported this outcome (24). Nusinersen cannot cross the blood-brain barrier and requires lifelong intrathecal injection for administration. It is distributed from the cerebrospinal fluid to the target central nervous system tissues, its safety remains a subject of ongoing debate (57). Lumbar puncture is an invasive medical procedure that carries the risk of adverse reactions, including headache, back pain, vomiting, infectious meningitis, and hydrocephalus (58).

In this meta-analysis, adverse reactions such as headache [15.17% (95% CI: 6.85%–25.61%)], back pain [13.92%(95% CI: 9.30%–19.19%)], and PLPS [9.59%(95% CI: 2.19%–20.63%) were attributed to the lumbar puncture procedure itself rather than nusinersen. Other common adverse reactions included nausea, respiratory tract infection, fever, abnormal liver function, pneumonia, dizziness, vomiting, and others. The main manifestations of adverse reactions were similar to those reported by Zhi-Juan Zhong et al. and Fu Zhengran et al. (56, 59). Fu Zhengran et al. (59) also reported findings based on real world data on adverse reactions of nusinersen gathered by the FDA. As for the safety outcomes, based on the published RWS in Chinese populations supported that the intrathecal administration of nusinersen was relatively safe and well-tolerated. No new safety concerns beyond the approved label were reported in the RWS of nusinersen.

There are several limitations to this meta-analysis. First, the included studies were real world observational studies, which are inherently subject to a relatively high risk of bias and lower quality. Second, a substantial degree of heterogeneity exists among some studies, which may be attributed to notable disparities in SMA type, SMN2 copy number, patient age, study duration, and other relevant factors. Due to the limited number of included studies, we were unable to conduct a meta-regression analysis on these factors, which may affect the accuracy of the results. Third, this meta-analysis only analyzed data on certain motor functions, including CHOP INTEND, HFMSE, and RULM at different follow-up times. Due to the limited data available, other motor function indicators, such as HINE-2 and 6MWT, were not analyzed at different follow-up times. In addition, the original data did not support separate meta-analyses for types 1, 2, 3, and 4, as only three of the 24 studies included in the meta-analysis reported motor function outcomes for type 4. Therefore, the primary analysis involved separate meta-analyses of types 2 and 3. Similarly, the original data did not support separate meta-analyses for children and adults, as the included studies reported motor function outcomes in both populations.

## Conclusions

This meta-analysis found that in RWS involving Chinese patients with SMA, nusinersen showed a significant improvement in motor function for Chinese patients with SMA types 1–4, which aligns with the findings from randomized trials. In studies with longer follow-up periods (≥14 months), Chinese SMA patients treated with nusinersen exhibited cumulative benefits in motor function indicators and clinical response rates. Regarding safety, no off-label safety concerns for nusinersen were observed. Common adverse events associated with intrathecal injection were consistent with the expected safety profile, and nusinersen sodium was generally well-tolerated. However, the majority of studies included in this review had a follow-up period of less than 24 months. Therefore, further long-term follow-up is necessary to assess the sustained therapeutic effects of nusinersen in Chinese patients with SMA.

## Data Availability

The original contributions presented in the study are included in the article/Supplementary Material, further inquiries can be directed to the corresponding author.
